# Leveraging Deep Learning to Construct a Programmed Cell Death-Driven Prognostic Signature in Acute Myeloid Leukemia

**DOI:** 10.3390/cimb48040354

**Published:** 2026-03-27

**Authors:** Chunlong Zhang, Haisen Ni, Ziyi Zhao, Ning Zhao

**Affiliations:** College of Computer and Control Engineering, Northeast Forestry University, Harbin 150040, China; zhangcl@nefu.edu.cn (C.Z.);

**Keywords:** acute myeloid leukemia, programmed cell death, deep learning, tumor immune microenvironment, prognostic signature

## Abstract

Acute myeloid leukemia (AML) is an aggressive hematologic malignancy characterized by profound molecular heterogeneity and high relapse rates, posing significant clinical challenges. Programmed cell death (PCD), encompassing diverse regulated modalities such as apoptosis, necroptosis, and ferroptosis, plays a key role in leukemogenesis and therapeutic response; however, a comprehensive prognostic framework integrating multi-modal PCD pathways in AML remains elusive. In this study, we performed a systematic transcriptomic analysis of 1624 genes associated with 13 distinct PCD forms. A novel computational pipeline combining a variational autoencoder (VAE) for dimensionality reduction and a multilayer perceptron (MLP) for classification was employed to identify robust PCD-related biomarkers, interpreted via SHapley Additive exPlanations (SHAP) analysis. This approach identified 48 candidate genes with discriminative potential between AML and normal bone marrow. Unsupervised consensus clustering based on these genes delineated two molecular subtypes exhibiting divergent clinical outcomes and immune microenvironment profiles. The subtype demonstrated an immunosuppressive phenotype, characterized by enriched regulatory T cells, M2 macrophages, and elevated expression of inhibitory immune checkpoints, correlating with inferior survival. We developed an 8-gene prognostic signature (*SORL1*, *PIK3R5*, *RIPK3*, *ELANE*, *GPX1*, *VNN1*, *CD74,* and *IL3RA*) that effectively categorized patients into high- and low-risk groups with notable survival differences, validated across independent cohorts. A prognostic nomogram combining the risk score, age, and cytogenetic risk enhanced the prediction accuracy for overall survival. Our study presents an integrative model that connects multi-modal PCD pathways to AML prognosis, offering a new molecular subtyping system and a clinically applicable risk assessment tool for improved prognostication and personalized treatment strategies.

## 1. Introduction

AML is an aggressive hematologic malignancy characterized by highly heterogeneous molecular abnormalities and the abnormal accumulation of immature myeloid progenitor cells in the bone marrow and peripheral blood [1]. According to Cancer Statistics, 2026, approximately 22,720 new cases of AML are expected to be diagnosed in the United States, with about 11,500 deaths projected in the same year, highlighting the high mortality burden associated with AML [2]. Despite notable progress in treating most hematologic malignancies with targeted therapies and combination strategies [3,4], treatment options for AML have remained relatively limited in recent decades. Therapeutic resistance and high relapse rate hinder AML patient survival [5,6], making the discovery of new prognostic biomarkers crucial for tracking disease progression and understanding its pathogenesis.

PCD is a genetically regulated process of cell death that plays a critical role in normal development and the maintenance of homeostasis in organisms. PCD is a complex process involving multiple mechanisms. It includes at least 13 distinct forms: apoptosis, pyroptosis, ferroptosis, autophagy, necroptosis, cuproptosis, parthanatos, entotic cell death, netotic cell death, lysosome-dependent cell death, alkaliptosis, oxeiptosis, and disulfidptosis [7]. Each form possesses unique characteristics: Apoptosis eliminates damaged or unnecessary cells [8]. Necroptosis is a regulated form of necrosis dependent on the activation of receptor-interacting protein kinases (RIPKs) [9]. Pyroptosis is defined by inflammatory cell death morphology, characterized by cell membrane pore formation, pro-inflammatory cytokine release, and cell lysis [10]. Ferroptosis is characterized by iron-dependent lipid peroxidation [11]. Cuproptosis is characterized by a copper-triggered modality [12]. Entotic cell death is characterized by invading living cells [13]. Netotic cell death involves the release of neutrophil extracellular traps [14]. Parthanatos is caused by the excessive excitation of the nuclease PARP-1 [15]. Lysosome-dependent cell death is facilitated by hydrolases, which permeate the cellular membrane and transport the contents to the cytosol [16]. Autophagy-dependent cell death is linked to a sequential process of lysosomal degradation, thereby facilitating metabolic adjustment and the recycling of nutrients [17]. Alkaliptosis is a novel form of PCD regulated by intracellular alkalinization [7,18]. Oxeiptosis is a unique PCD pathway that orchestrates cell death by leveraging the reactive oxygen sensing capabilities of *KEAP1*, potentially functioning in concert with other PCD pathways [7,19].

The dysregulation of PCD is a hallmark of cancer, enabling tumor cells to evade elimination and resist therapy. Each has distinct genetic regulators and implications in oncogenesis. Experimental evidence has steadily unveiled the complex roles these pathways play in various cancers. Apoptosis is frequently inactivated in cancers through mutations in *TP53* or the overexpression of anti-apoptotic proteins like BCL-2, a well-documented phenomenon in chronic lymphocytic leukemia [20]. Necroptosis, a regulated necrotic process, can be suppressed in cancers through the downregulation of key effector *RIPK3* [21], and its restoration has been shown to induce cell death in AML models [22]. The tumor-suppressive role of pyroptosis is mediated by the cleavage of *GSDME* by caspase-3, the epigenetic silencing of *GSDME* is frequently observed in gastric cancer, and its re-expression sensitizes cells to chemotherapeutic drugs [23]. Ferroptosis, driven by iron-dependent lipid peroxidation, is regulated by the cystine/glutamate antiporter *SLC7A11* (xCT). Inhibiting *SLC7A11* induces ferroptosis and suppresses tumor growth in vivo, highlighting its therapeutic potential [24]. The newly identified cuproptosis links copper-dependent cell death to mitochondrial metabolism, where the aggregation of lipoylated proteins like *DLAT* and subsequent proteotoxic stress, orchestrated by the key regulator *FDX1*, can be targeted in cancers with high oxidative phosphorylation [25]. Furthermore, parthanatos, dependent on hyperactivation of *PARP1*, contributes to neuronal death and is exploited in cancer therapy with *PARP* inhibitors [26]. The process of entosis, governed by RhoA-ROCK signaling and adherens junctions, is commonly observed in human breast cancers and can lead to cell competition [27,28]. NETosis, dependent on PAD4-mediated histone citrullination, has been implicated in forming metastasis-promoting niches in models of liver metastasis [29]. Lysosome-dependent cell death can be triggered by lysosomotropic agents that destabilize the lysosomal membrane, a process involving cathepsins, and is a mechanism of action for drugs like siramesine in breast cancer cells [30]. While autophagy often promotes tumor cell survival under stress, excessive autophagy can lead to autophagy-dependent cell death, as demonstrated by the degradation of the pro-survival protein BCL-2 in response to certain agents [31]. Emerging pathways like alkaliptosis, which is potentiated by the inhibition of *CA9* in pancreatic cancer models [18], and oxeiptosis, a ROS-induced but non-inflammatory death mediated by the KEAP1-PGAM5-AIFM1 axis, further expand the repertoire of cell death mechanisms with potential therapeutic applications [19]. Finally, disulfidptosis, a recently described form of death triggered by glucose starvation in SLC7A11-high cells, leads to aberrant disulfide bond formation and collapse of the actin cytoskeleton, revealing a metabolic vulnerability in cancers [32]. This intricate network of PCD pathways, governed by specific genetic and molecular regulators, not only deepens our understanding of tumor biology but also unveils a vast landscape of potential targets for innovative cancer therapeutics.

Although PCD has been extensively studied in solid tumors, its mechanisms and prognostic value in AML remain underexplored. Systematic research on different forms of PCD and their regulatory factors in AML is still relatively scarce.

In this study, we developed a deep learning-integrated framework to construct and validate a PCD-relevant prognostic signature for AML. This risk model demonstrated robust predictive power and clinical independence, culminating in the identification of a novel PCD-based AML subtype with potential implications for personalized prognosis and therapy.

## 2. Materials and Methods

### 2.1. Data Collection and Processing

Genes associated with the 13 PCD patterns were sourced from the Gene Set Enrichment Analysis (GSEA), the Kyoto Encyclopedia of Genes and Genomes (KEGG) database, and related literature. The PCD patterns include apoptosis, pyroptosis, ferroptosis, autophagy, necroptosis, cuproptosis, parthanatos, entotic cell death, netotic cell death, lysosome-dependent cell death, alkaliptosis, oxeiptosis, and disulfidptosis [7,33,34,35]. The TCGA-LAML dataset was downloaded from the TCGA database (https://portal.gdc.cancer.gov/) [36]. After excluding duplicate samples, those lacking clinical information, and those with zero survival time, raw transcriptomic data and corresponding clinical information from 151 AML samples were ultimately included. Since the TCGA database did not include normal samples for AML, we collected 70 normal bone marrow (BM) samples from the GTEx database (http://www.GTExportal.org/home/). Additionaly, two AML datasets (GSE10358 n = 92, GSE71Sample Number 14 n = 105) were Sample Numberollected to validate the LASSO Cox results from the GEO database [37,38].

### 2.2. Differential Gene Expression Analysis

Differential gene expression analysis was conducted by comparing GTEx normal blood samples with AML samples using the limma package in R 3.62.2 [39]. The cutoff criteria for differentially expressed genes (DEGs) were set at |Log_2_FC| ≥ 1 and *p* value ≤ 0.05.

### 2.3. AML Biomarker Identification Model

To identify robust PCD-related biomarkers, we developed a hybrid deep learning framework integrating a VAE for dimensionality reduction and an MLP for classification, followed by SHAP-based model interpretation. All modeling and interpretation procedures were implemented in Python 3.8, using PyTorch 1.10.0 for model construction and training, scikit-learn 1.3.0 for data splitting, normalization, and performance evaluation, SHAP 0.44.1 for model interpretation, and pandas 2.0.3 and numpy 1.24.3 for data processing. The VAE component served as a critical generative model for robust feature representation. Its encoder consisted of three fully connected layers with 256 and 64 neurons, ultimately outputting the mean and log-variance to form a 64-dimensional latent space. A symmetric decoder was designed to reconstruct the original input from these latent variables. Subsequently, the extracted latent features were fed into the MLP classifier. The MLP comprised three fully connected layers containing 32, 16, and 1 neurons respectively, utilizing a sigmoid activation function in the final layer to output the predicted probability of a sample being tumorous.

The VAE was used to learn a compact latent representation of gene expression data. The encoder consisted of three fully connected layers (input dim → 256 → 64 → latent parameters), outputting the mean and log-variance of the latent distribution:

(1) $$\mu ,log\sigma^{2}=Encoder(x)$$

where $x\in R^{d}$ represents the input gene expression vector with d genes, $\mu \in R^{k}$ and $\log\sigma^{2}\in R^{k}$ denote the mean and log-variance vectors of the latent space, and k is the latent dimension. Latent variables were sampled using the reparameterization trick:

(2) z=μ+σ⊙ϵ,ϵ∼N(0,I)

where $z\in R^{k}$ is the latent vector, $\sigma \;=\;\exp\left(\frac{1}{2}\log\sigma^{2}\right)$ is the standard deviation, ϵ is a random vector sampled from a standard normal distribution, and ⊙ denotes element-wise multiplication. The decoder reconstructed the input data:

(3) $$\hat{x}=\mathrm{Decoder}(z)$$

where $\hat{x}$ represents the reconstructed gene expression vector.

The latent dimension was set to 32, and the decoder mirrored the encoder structure (32 → 64 → 256 → input dim) with a sigmoid activation function applied to reconstruct normalized expression values. The MLP classifier took the latent vector zas input and consisted of three fully connected layers (32 → 32 → 16 → 1), with ReLU activation in hidden layers and a sigmoid activation function in the output layer to produce the probability of a sample being tumorous:

(4) $$\hat{y}=MLP(z)=\sigma (W_{2}\cdot ReLU(W_{1}\cdot z+b_{1})+b_{2})$$

where $W_{1},W_{2}$ are weight matrices, $b_{1},b_{2}$ are bias terms, and $\hat{y}\in [0,1]$ represents the predicted probability of the sample belonging to the tumor class.

Gene expression data were normalized using Min-Max scaling before being input into the model. Samples were randomly divided into training and test sets at a ratio of 8:2, and the procedure was repeated five times with different random seeds to assess model robustness. The model was trained for 250 epochs using the Adam optimizer with a learning rate of 0.001. The total loss function consisted of reconstruction loss, KL divergence, and classification loss:

(5) $$L_{total}\;=\;{BCE}_{\mathrm{reconstruction}}\;+\;{KL}_{\mathrm{divergence}}\;+\;\lambda \cdot {BCE}_{\mathrm{classification}}$$

where λ = 0.001. The KL divergence term was defined as:

(6) $$\mathrm{KL}=-\frac{1}{2}\sum_{j=1}^{k}\left(1+log\sigma_{j}^{2}-\mu_{j}^{2}-\sigma_{j}^{2}\right)$$

where k is the latent dimension and j indexes each latent variable.

To quantify the contribution of each gene feature to model predictions, SHAP analysis was performed using the KernelExplainer. The prediction function f(x) consisted of VAE encoding followed by MLP classification. The SHAP value for each feature was calculated as:

(7) $$\phi_{j}=\sum_{S\subseteq \{1,\ldots ,d\}\setminus \{j\}}\frac{|S|!(d-|S|-1)!}{d!}\left[f(S\cup \{j\})-f(S)\right]$$

where $\phi_{j}$ represents the contribution of feature j, S is a subset of features excluding j, and f(⋅) denotes the model prediction function.

The resulting SHAP value matrix was defined as:

(8) $$\mathrm{SHAP}\;\mathrm{Value}\;\mathrm{Matrix}\;\in R^{n_{test}\times d}$$

where $n_{\mathrm{test}}$ is the number of test samples and d is the number of input genes.

The SHAP output matrix was structured with rows corresponding to each sample and columns representing the contribution value of each gene, where positive values indicated positive contributions to predictions and negative values denote negative impacts [40]. The Biomarker Potential Score mechanism employed a scoring function where the directional contribution of SHAP values was quantified by calculating the absolute difference between mean SHAP values in tumor versus normal samples [41]. For each gene, define:

(9) $$P_{g}^{+}=\frac{Number\;of\;tumor\;samples\;with\;SHAP\;values>0}{Total\;number\;of\;tumor\;samples}$$

(10) $$P_{g}^{-}=\frac{Number\;of\;normal\;samples\;with\;SHAP\;values<0}{Total\;number\;of\;normal\;samples}$$

The final scoring formula adopted the form of a harmonic mean:

(11) $${Score}_{g}=\left\{\begin{array}{ll} \frac{2\cdot P_{g}^{+}\cdot P_{g}^{-}}{P_{g}^{+}+P_{g}^{-}}, & \;\;\;\;if\;P_{g}^{+}+P_{g}^{-}\neq 0\\ 0, & \;\;\;\;else \end{array}\right.$$

The ideal biomarker gene should exhibit the following characteristics: Positive contribution to predictions in tumor samples (SHAP value > 0); and negative contribution to predictions in normal samples (SHAP value < 0). This pattern indicated that the gene has discriminatory power between the two states, making it a potential diagnostic or prognostic biomarker.

### 2.4. Functional Enrichment Analysis

To characterize the biological activities and signaling pathways enriched in the target gene list, Gene Ontology (GO) analysis was performed using the clusterProfiler package in R 4.18.4 covering Biological Process (BP), Molecular Function (MF), and Cellular Component (CC) categories [42]. Additionally, KEGG enrichment analysis was conducted to identify significantly enriched pathways. A functional enrichment threshold of *p* value ≤ 0.05 based on hypergeometric testing was considered statistically significant.

### 2.5. Single-Sample Gene Set Enrichment Analysis

To evaluate pathway activity differences between AML subtypes, single-sample gene set enrichment analysis (ssGSEA) was performed using the GSVA R package in R 2.4.8 [43,44]. Gene sets were obtained from the Molecular Signatures Database (MSigDB, HALLMARK collection), which summarizes well-defined biological states and processes. The ssGSEA transforms the gene expression matrix into a pathway activity matrix by computing enrichment scores based on the ranked gene expression levels per sample. Finally, differences in pathway activities between subtypes were compared using the Wilcoxon rank-sum test, with statistical significance defined by an adjusted *p* value ≤ 0.05.

### 2.6. Molecular Subtyping of AML Patients

Based on the expression levels of the PCD-related marker genes closely associated with AML, consensus clustering was performed using the ConsensusClusterPlus package in R 1.74.0 to identify distinct AML subtypes [45]. To ensure classification stability, the “re-sampling times” parameter was set to 1000. Kaplan–Meier (KM) analysis was conducted using the survminer package in R 0.5.1, with a statistical significance threshold set at *p* value ≤ 0.05.

### 2.7. Characterization of the Tumor Immune Microenvironment Across PCD Subtypes

To evaluate differences in immune infiltration levels among distinct PCD subtypes, we employed CIBERSORT and xCELL to estimate immune cell infiltration levels [46,47], followed by comparison of differences between subtypes using the Wilcoxon test. Additionally, expression levels of inhibitory feature genes were compared across subtypes. Human leukocyte antigen (HLA) molecules played essential roles in immune regulation, including antigen presentation and activation of T-helper cells [48]. In this study, we further compared the expression profiles of HLA molecules across different molecular subtypes. The threshold of *p* value ≤ 0.05 was applied for analysis.

### 2.8. Drug Response Correlation Evaluation

To assess drug efficacy, the oncoPredicR package in R 4.4.3 (https://github.com/HuangLabUMN/oncoPredict, accseed on 15 January 2025) was employed as a robust tool for predicting drug sensitivity [49]. Differences in drug sensitivity correlations between subtypes were compared using the Wilcoxon test. The threshold of *p* value ≤ 0.05 was applied for analysis.

### 2.9. Construction and Validation of a PCD-Related Prognostic Signature for AML Patients

Univariate Cox regression analysis was used to screen for overall survival (OS)-related DEGs. A risk signature was established via Cox regression with the least absolute shrinkage and selection operator (LASSO) penalty through the glmnet package in R 4.1.10 [49]. The minimum partial likelihood deviance was utilized to determine the model’s penalty parameter (λ). The regression coefficients (β) from the LASSO model were linearly combined with gene expression levels to calculate the prognostic risk score:

(12) $$\mathrm{Risk}\;\mathrm{score}=\sum_{i}^{n}{Coef}_{i}\times A_{i}$$

AML patients were stratified into high-risk and low-risk groups based on the median risk score. The predictive power of the risk-based prognostic signature was evaluated using KM survival analysis and time-dependent receiver operating characteristic (ROC) curve analysis [50].

To validate the predictive accuracy of the risk signature, two external GEO datasets—GSE10358 (92 samples) and GSE71014 (105 samples)—were downloaded as independent validation sets. The signature was applied to calculate the risk score for each patient, and KM curves were generated to assess its performance in predicting survival outcomes.

### 2.10. Construction of a Prognostic Nomogram for AML Patients

Based on clinical information of AML patients (age, gender, cytogenetic risk (CR), and French-American-British classification (FAB)) and the risk score, univariate and multivariate Cox regression analysis were performed. A prognostic nomogram was constructed using the survival and rms packages in R (v3.8.3, v8.1.0). The accuracy of the nomogram was evaluated using ROC curves and the concordance index (C-index). CR was a prognostic framework that stratified patients into favorable, intermediate, or adverse risk groups based on chromosomal abnormalities in leukemia cells, such as translocations or deletions. This system was pivotal for guiding treatment intensity decisions. Separately, the FAB Classification represented a morphological typing system, which categorized AML into M0 through M7 subtypes according to cellular morphology and lineage differentiation observed under microscopy [51].

## 3. Result

### 3.1. Potential PCD Gene Biomarkers in AML

**Figure 1:**
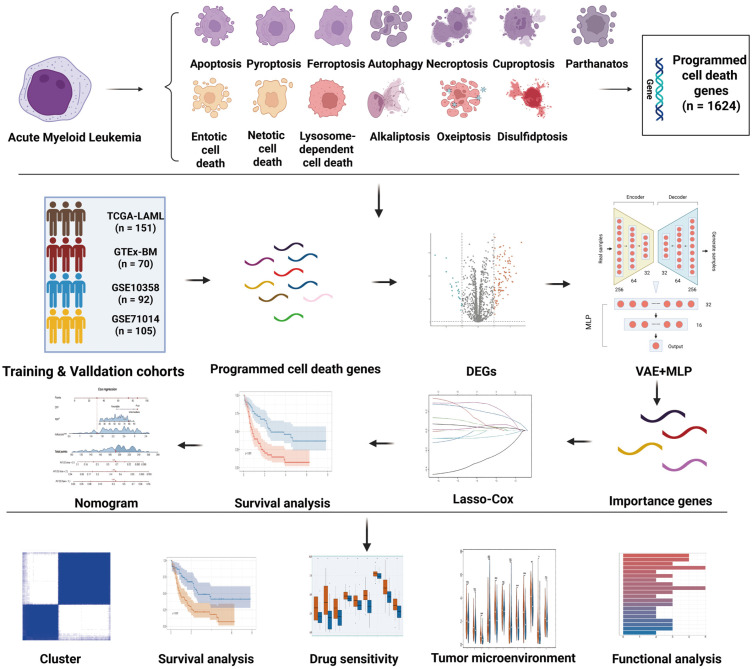
Flowchart for comprehensive analysis of diverse cell death patterns in patients with AML.

**Figure 2:**
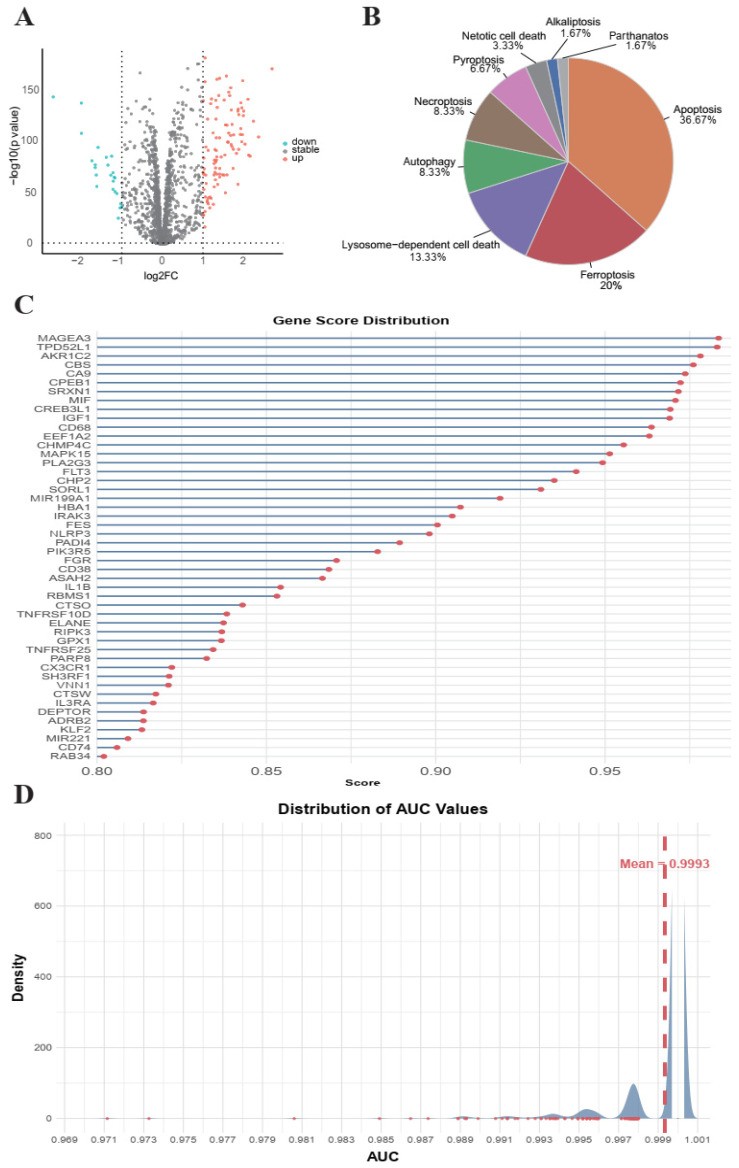
Screening of PCD-related key biomarkers in AML. (**A**) Differential expression analysis of PCD-associated genes in AML. The red and blue color represent the significantly up-regulated and down-regulated genes. The X-axis represents the log2 fold change (log2FC), and the Y-axis represents the −log10 (*p* value) from the differential expression analysis. (**B**) Number of candidate genes identified in each PCD modality. (**C**) Biomarker potential scores of the 48 candidate genes SHAP analysis. (**D**) AUC value of the trained prediction model. The X-axis represents the AUC value, and the Y-axis represents density.

To identify robust PCD-related biomarkers in AML, we executed a sequential analytical workflow (Figure 1). We initially compiled a global set of 1624 genes across 13 PCDs. From this comprehensive set, we screened for AML-related DEGs by integrating 151 TCGA-AML samples and 70 GTEx normal bone marrow samples. Using the criteria of *p* value ≤ 0.05 and |log2FC| ≥ 1, a total of 124 DEGs were identified (Figure 2A). The expression matrix of these 124 DEGs was subsequently processed through a hybrid VAE-MLP deep learning model. A staged training strategy was adopted: The first 50 epochs involved unsupervised pre-training of the VAE module to learn a stable latent feature space. Epochs 51–100 involved training of the MLP classifier. Epochs 101–250 involved joint training of VAE and MLP to optimize both reconstruction and classification performance. This model achieved exceptional classification performance on the testing set with an AUC > 0.99 (Figure 2D). Ultimately, SHAP analysis was applied to interpret the model’s predictions. By evaluating the marginal contribution of each gene and applying an importance score threshold > 0.8, we retained 48 core candidate genes. These genes demonstrated stable and significant contributions to the classification task, highlighting their potential research value as biomarkers for AML (Figure 2B,C).

### 3.2. The Functional Enrichment Analysis of PCD Marker Genes

**Figure 3:**
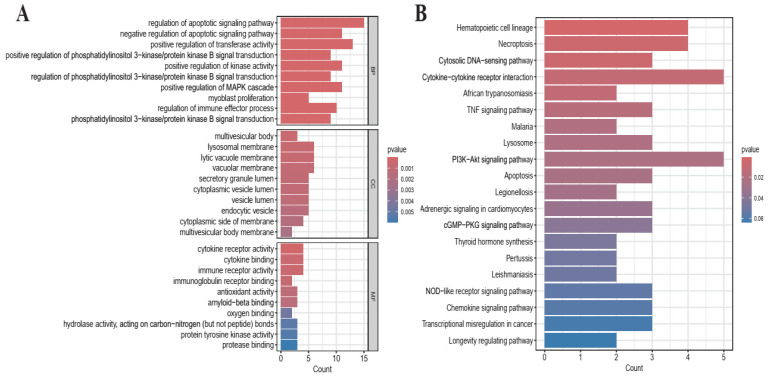
Functional enrichment analysis of the 48 candidate genes. (**A**) GO enrichment analysis. The top barplot represents the result of BP. The middle barplot represents the result of CC. The bottom barplot represents the result of MF. (**B**) KEGG enrichment analysis. The count of each barplot represents the number of genes enriched in function, and the color represents the *p* value.

To further elucidate the biological processes and signaling pathways involving these PCD genes, functional enrichment analysis was performed using the 48 candidate genes. GO analysis categorized gene functions into BP, MF, and CC. Our results revealed significant enrichment in BP and MF terms closely associated with cell death regulation and immune response (Figure 3A). In BP, genes were significantly enriched in regulation of apoptotic signaling pathways, immune effector processes, and regulation of PI3K/AKT and MAPK signaling pathways. These processes indicate that the identified genes are not only involved in programmed cell death but also actively participate in immune regulation and key oncogenic signaling pathways [52,53]. In CC, genes were significantly enriched in lysosomal membrane, vesicle lumen, and secretory granule structures, which suggests a potential link between cell death mechanisms and intracellular trafficking or lysosome-mediated processes [54]. In MF, genes were enriched in cytokine receptor activity, immune receptor binding, and protein kinase activity, which support the involvement of these genes in immune signaling and signal transduction [55,56].

KEGG analysis mapped genes into known and larger-scale signaling pathways, demonstrating their coordinated action in established disease-relevant networks. PCD genes were extensively involved in several critical pathways (Figure 3B). The most prominently enriched pathway was Hematopoietic cell lineage, strongly affirming the central role of these genes in blood cell development and the specific pathogenesis of AML [57]. Beyond classical necroptosis and apoptosis, the genes were significantly enriched in immune-inflammatory pathways, including cytokine–cytokine receptor interaction, TNF signaling pathway, and cytosolic DNA-sensing pathway. This extensive enrichment strongly suggests that these biomarkers reflect the dynamic crosstalk between PCD and the AML immune microenvironment [58,59]. Furthermore, enrichment in pathways such as hematopoietic cell lineage and PI3K–Akt signaling pathway indicates involvement in hematopoietic differentiation and survival signaling, both of which are central to leukemia development [60,61].

This integrated functional profile demonstrates that the dysregulation of these PCD-related pathways in AML extends beyond isolated cellular events. It drives a vicious cycle of aberrant cell proliferation, immune evasion, and chemoresistance and relapse. Consequently, our 48-gene signature captures a core pathogenic network, providing a robust mechanistic foundation for its prognostic power and highlighting potential therapeutic vulnerabilities in AML.

### 3.3. Molecular Subtypes of AML Based on PCD

**Figure 4:**
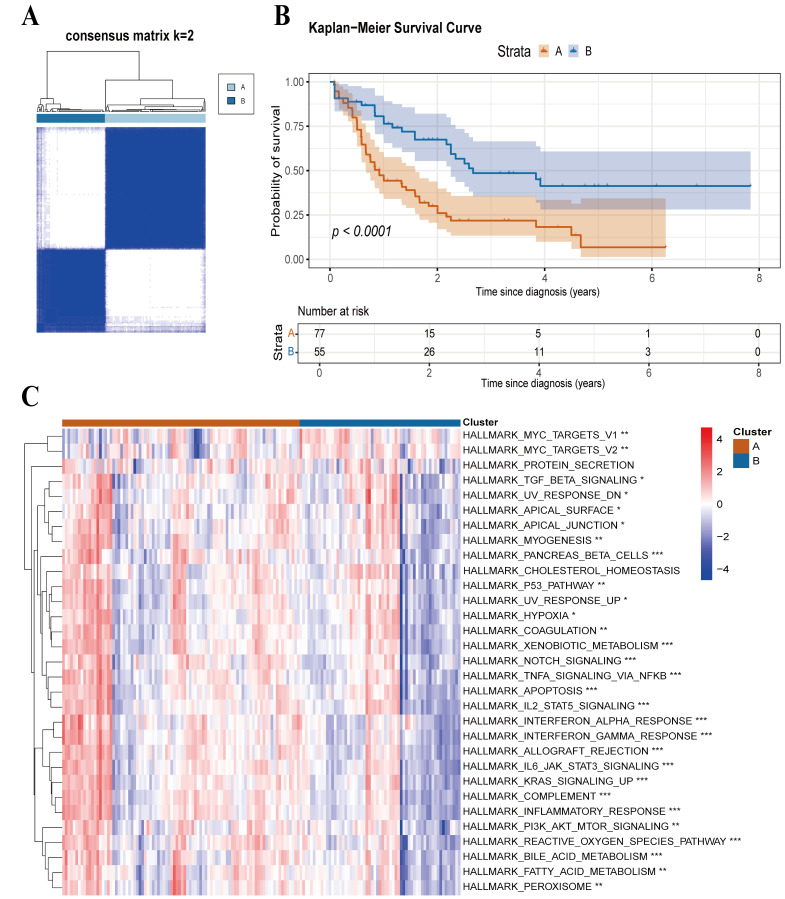
Identification of molecular subtypes via consensus clustering. (**A**) Consensus clustering analysis determining two stable clusters (k = 2). (**B**) Kaplan–Meier survival curves for Subtype A versus Subtype B; the log-rank test was used to assess statistical significance. (**C**) Heatmap of ssGSEA scores comparing pathway enrichment profiles between the two subtypes. Statistical significance is denoted as follows: “***”, “**” and “*” denote *p*-values of ≤0.001, 0.01, and 0.05. Differences were compared using the Wilcoxon rank-sum test.

To explore PCD-associated molecular heterogeneity, we applied consensus clustering to categorize AML samples based on prognostic PCD gene profiles. The K-means algorithm identified two stable subtypes, Subtype A and Subtype B (Figure 4A). Notably, patients in Subtype A had significantly shorter OS than those in Subtype B, suggesting substantial differences in their underlying PCD-related mechanisms (Figure 4B).

To elucidate the functional disparities between the subtypes, we applied ssGSEA to quantify HALLMARK pathway enrichment (Figure 4C). Notably, Subtype A exhibited significantly elevated enrichment in immune-inflammatory pathways, specifically the interferon and inflammatory response, compared to Subtype B. These findings suggest that PCD-related genes not only influence AML prognosis but also actively drive immune microenvironment remodeling and disease stratification.

### 3.4. Characteristics of the Tumor Immune Microenvironment in PCD Subtypes

Functional enrichment of PCD-related genes revealed robust associations with immune regulation pathways, suggesting that PCD actively modulates both AML development and the tumor immune microenvironment (TIME). Prompted by these insights, we characterized TIME heterogeneity between Subtypes A and B to uncover potential immune escape mechanisms and differences in immunotherapy responses.

**Figure 5:**
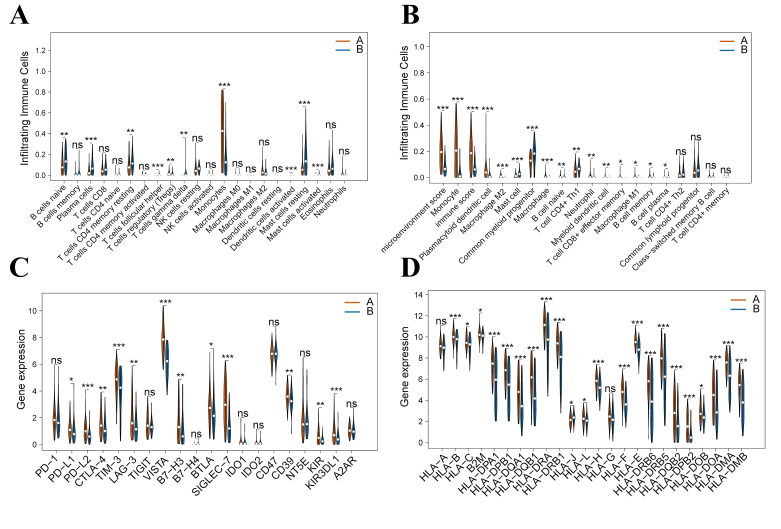
Characterization of the tumor immune microenvironment (TIME) across defined subtypes. (**A**) Immune cell infiltration profiles in Subtype A versus B, deconvoluted using CIBERSORTx in the TCGA–AML cohort. (**B**) Immune cell composition in Subtype A versus B, assessed with the xCell algorithm. (**C**) Expression patterns of inhibitory immune checkpoint molecules between the two subtypes. (**D**) Expression levels of human leukocyte antigen (HLA) genes across subtypes. Statistical significance is denoted as follows: “***”, “**”, “*” and “ns” denote *p* values of ≤0.001, 0.01, 0.05, and not significant. Differences were assessed using the Wilcoxon rank-sum test.

To systematically profile immune cell infiltration across the subtypes, we employed established deconvolution algorithms, including CIBERSORTx and xCell, to ensure robust characterization (Figure 5).

CIBERSORTx analysis showed that Subtype B significantly exhibited infiltration of naive B cells, activated CD4 memory T cells, and activated mast cells, indicative of a more robust anti-tumor immune response. In contrast, Subtype A was markedly enriched in regulatory T cells (Tregs), resting mast cells, and gamma delta T cells. This distinct infiltration pattern underscores a predominantly immunosuppressive microenvironment in Subtype A (Figure 5A).

Subsequent xCell analysis corroborated these findings, revealing that Subtype A exhibited elevated infiltration of monocytes and M2 macrophages, both linked to immunosuppression and tumor promotion. Conversely, CD8+ T cells and CD4+ Th1 cells were markedly enriched in Subtype B, reflecting a robust cytotoxic immune response (Figure 5B).

To evaluate immunotherapeutic potential, we profiled the expression of immune checkpoint molecules (Figure 5C). Notably, a broad panel of inhibitory immune checkpoint molecules (including PD-L1, PD-L2, CTLA-4, TIM-3, LAG-3, VISTA, B7-H3, CD39, KIR, etc.) was significantly upregulated in Subtype A, suggesting a more immunosuppressive microenvironment that might lead to immune evasion and impaired antigen presentation.

Robust HLA-mediated antigen presentation is fundamental to anti-tumor immunity. Paradoxically, we found that most HLA genes were significantly upregulated in the immunosuppressive Subtype A (Figure 5D). This upregulation likely reflects a compensatory response driven by the interferon-enriched inflammatory microenvironment characteristic of this subtype. In Subtype A, the potent co-expression of inhibitory checkpoints (e.g., PD-L1 and CTLA-4) effectively attenuated HLA-mediated T-cell responses, counteracting its heightened antigen presentation readiness. This concurrent upregulation of HLA molecules and inhibitory checkpoints defines a profoundly exhausted immune microenvironment, where the immune system is primed for activation yet functionally blunted, ultimately failing to mount an effective anti-leukemic response.

Collectively, these findings indicate that Subtype A features a dysfunctional TIME, driven by limited immune activation and enhanced checkpoint inhibition, constraining endogenous anti-tumor immunity. Importantly, this pronounced immunosuppressive phenotype suggests that Subtype A may be particularly responsive to immune checkpoint inhibitors (ICIs), highlighting a critical therapeutic vulnerability.

In summary, our study not only delineates profound immune microenvironment heterogeneity between PCD subtypes but also provides a robust framework for developing personalized immunotherapy strategies, particularly for identifying ICI-sensitive populations.

### 3.5. Drug Sensitivity and Treatment Strategy in PCD Subtypes

**Figure 6:**
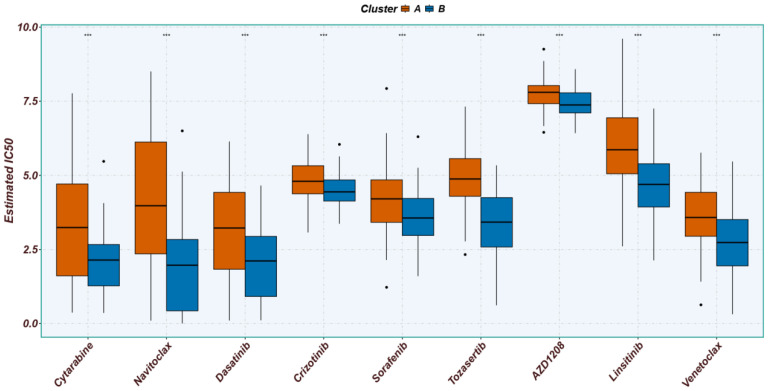
Correlation between the two subtypes and drug sensitivity in AML patients. The X-axis represents commonly used therapeutic agents for leukemia, and the Y-axis represents the IC50 value. Statistical significance is denoted as follows: “***” denote *p* values of ≤0.001.

To evaluate subtype-specific pharmacological vulnerabilities, we estimated the half-maximal inhibitory concentrations (IC_50_) of standard AML therapeutics (Figure 6). Notably, Subtype B exhibited significantly lower IC_50_ values for key agents (e.g., Cytarabine, Navitoclax, Dasatinib, and Venetoclax) [62,63,64], reflecting heightened drug sensitivity. Conversely, the higher IC_50_ values in Subtype A indicate a pronounced chemoresistant phenotype, aligning with their poorer prognosis and suboptimal responses to conventional regimens.

Collectively, these findings indicate that PCD-based subtyping not only delineates molecular heterogeneity in AML but also reflects profound subtype-specific pharmacological vulnerabilities. Given their heightened chemosensitivity, Subtype B patients are likely to derive greater clinical benefit from standard induction regimens. Conversely, the intrinsic chemoresistance of Subtype A necessitates alternative therapeutic modalities, such as combinatorial targeted approaches or immunotherapy, to overcome therapeutic bottlenecks.

Ultimately, these distinct drug sensitivity profiles provide a powerful translational framework for precision oncology in AML. By stratifying patients based on these molecular signatures, clinicians can optimize therapeutic efficacy while minimizing futile toxicities, thereby improving overall clinical outcomes.

### 3.6. Construction and Validation of Prognostic Features Associated with PCD Subtypes

**Figure 7:**
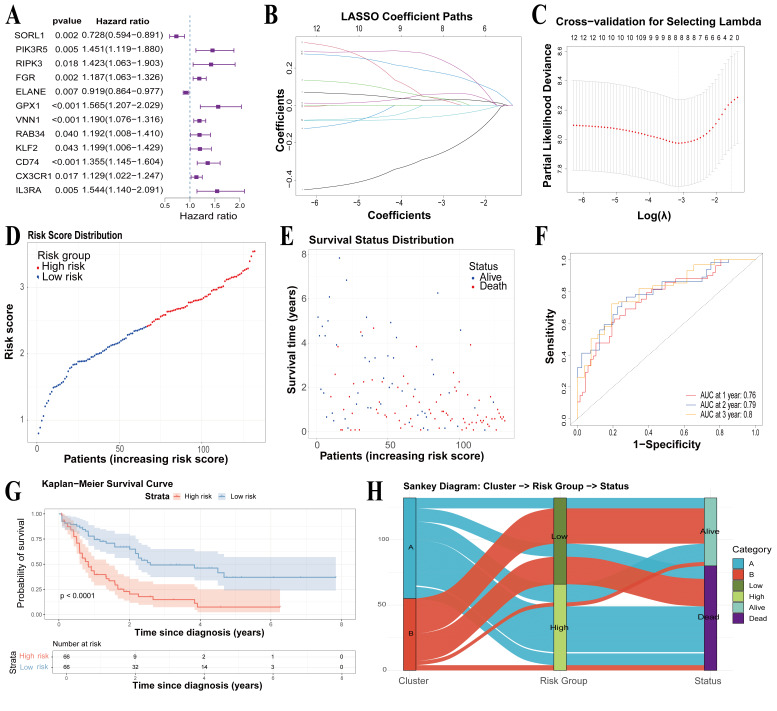
Construction of a risk-signature model in the TCGA-AML cohort. (**A**) Univariate Cox regression analysis of the 48 candidate genes. The forest plot displays the 12 genes significantly associated with survival (*p* value < 0.05). (**B**) LASSO-Cox regression analysis of the 12 survival-related genes. (**C**) Cross-validation for selecting the optimal tuning parameter (λ) in the LASSO regression. (**D**) Distribution of AML patients based on calculated risk scores. (**E**) Survival status distribution of AML patients. (**F**) Time-dependent ROC curves demonstrating the predictive efficiency of the risk-signature model. (**G**) Kaplan–Meier survival curves for the high-risk and low-risk groups. (**H**) Alluvial plot illustrating the relationships among molecular subtype, risk group, and survival status.

To translate the observed TIME heterogeneity into a clinically applicable prognostic tool, we first evaluated the 48 PCD-related genes using univariate Cox regression to identify candidate genes significantly associated with OS (Figure 7A). Subsequently, these prognostically relevant candidates were subjected to LASSO Cox regression. By employing LASSO to penalize and eliminate redundant features, this approach minimized overfitting and successfully constructed a robust, highly representative risk signature. Based on the optimal regularization parameter λ selected via cross-validation, a risk signature model comprising eight key genes (*SORL1*, *PIK3R5*, *RIPK3*, *ELANE*, *GPX1*, *VNN1*, *CD74*, and *IL3RA*) was ultimately established (Figure 7B,C). The prognostic risk score for each patient was calculated using the following formula: Risk Score = (−0.2538 × *SORL1* expression) + (0.0294 × *PIK3R5* expression) + (0.0370 × *RIPK3* expression) + (−0.0572 × *ELANE* expression) + (0.0614 × *GPX1* expression) + (0.0291 × *VNN1* expression) + (0.1725 × *CD74* expression) + (0.2428 × *IL3RA* expression). Here, a positive β coefficient indicated that high expression of the gene was associated with poor prognosis, and a negative coefficient represented a protective factor.

Patients were stratified into high-risk and low-risk groups based on the median risk score. KM survival analysis revealed that the high-risk group had a significantly shorter OS than their low-risk counterparts (*p* value < 0.001) (Figure 7D,G). The number of deaths increased with the increasing risk score, demonstrating the strong discriminatory power of the model in AML prognosis assessment (Figure 7D,E). Time-dependent ROC analysis further showed robust predictive accuracy, with AUC values of 0.76, 0.79, and 0.80 for 1-year, 2-year, and 3-year survival, respectively (Figure 7F). Additionally, an alluvial plot showed that most Subtype A patients were in the high-risk group, whereas Subtype B patients were mainly in the low-risk group (Figure 7F). This further confirmed that the risk model not only serves as a powerful prognostic indicator but also faithfully captures the underlying immunological heterogeneity.

**Figure 8:**
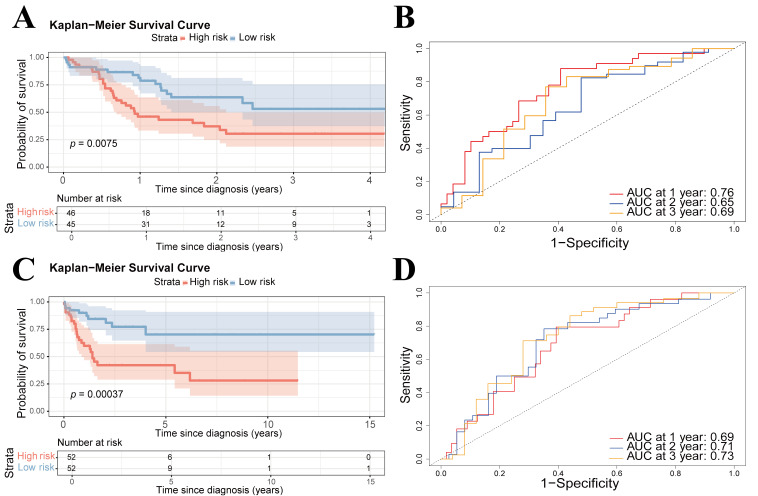
Validation of the risk-score model in external datasets. (**A**) Kaplan–Meier curves for high- and low-risk groups in the GSE10358 cohort. (**B**) ROC curves of the prognostic signature at 1-, 2-, and 3-year time points in GSE10358. (**C**) Kaplan–Meier curves for high- and low-risk groups in the GSE71014 cohort. (**D**) ROC curves of the prognostic signature at 1-, 2-, and 3-year time points in GSE71014.

To assess the generalizability and robustness of the eight-gene signature, we validated its prognostic performance across two independent AML cohorts, GSE10358 and GSE71014. Based on the risk score formula derived from the training set, patients in the testing sets were stratified into high-risk and low-risk groups. In GSE10358, high-risk patients exhibited a significantly shorter OS than low-risk patients (*p* value = 0.00037) (Figure 8A). A similar prognostic stratification was successfully achieved in GSE71014 (*p* value = 0.0075) (Figure 8C), strongly affirming the cross-cohort reproducibility and broad applicability of our model.

Consistent with the training cohort, patient mortality across all external validation sets escalated proportionally with increasing risk scores, confirming the signature as a robust determinant of survival outcomes. ROC curve analysis validated the model’s predictive performance in external cohorts, with AUC values around 0.7 for 1-year, 2-year, and 3-year survival, indicating moderate to good predictive capability across different datasets (Figure 8B,D). This sustained performance underscores the model’s exceptional generalizability and its tremendous potential for clinical translation.

### 3.7. The Prognostic Risk Score Identified as an Independent Predictor in AML

**Figure 9:**
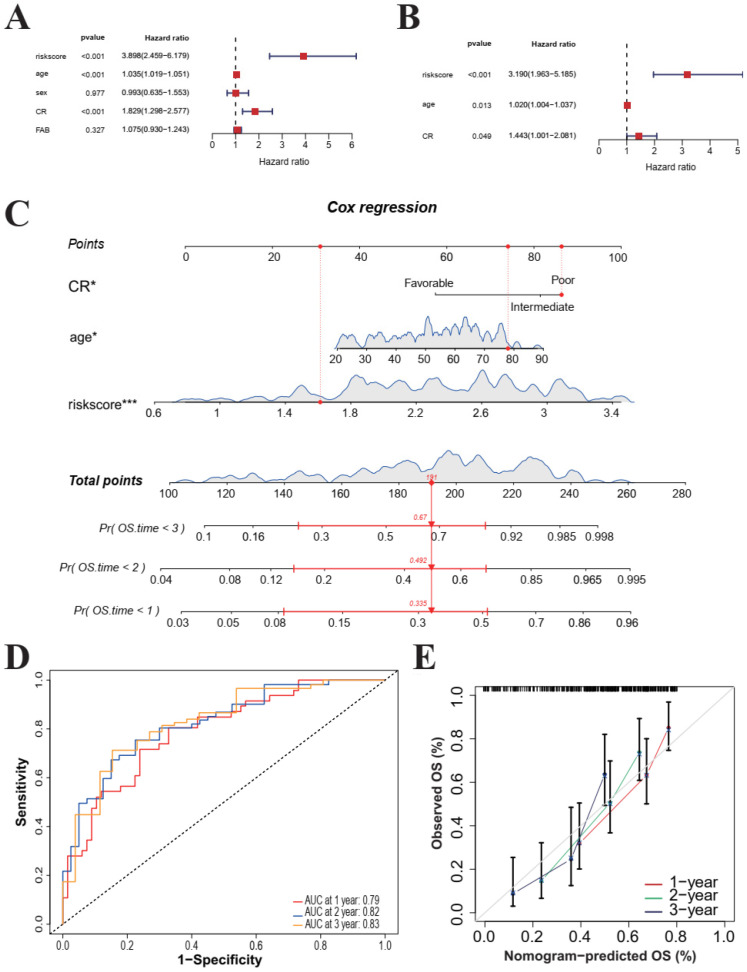
Development and assessment of a prognostic nomogram model. (**A**) Univariate analysis of clinicopathological features and the risk score in the TCGA cohort. (**B**) Multivariate analysis of clinicopathological features and the risk score in the TCGA cohort. (**C**) Construction of a nomogram for predicting the prognosis of AML patients. (**D**) Receiver operating characteristic (ROC) curve analysis for the TCGA cohort. (**E**) Calibration plots illustrating the concordance between predicted and observed 1-, 2-, and 3-year overall survival probabilities in the TCGA cohort. “***” and “*” denote *p*-values of ≤0.001, and 0.05.

To establish a comprehensive and clinically actionable prognostic model, we subjected the risk score and standard clinical variables (including age, gender, CR, and FAB subtype) to univariate and multivariate Cox regression analyses (Figure 9A).

Notably, the risk score maintained its statistical significance (*p* value ≤ 0.05) in the multivariate Cox regression analysis, confirming its role as an independent prognostic factor. Additionally, age and CR were identified as independent predictors of survival (Figure 9B). These findings highlight the value of integrating clinical and molecular information to enhance the precision of AML prognostication.

Based on these multivariate results, we constructed a comprehensive nomogram, incorporating the risk score, age, and cytogenetic risk to provide clinicians with an intuitive tool to estimate survival probabilities at 1, 2, and 3 years (Figure 9C). In this nomogram, the risk score carried the highest weight, exerting the dominant influence on survival predictions, followed by cytogenetic risk and age. This further underscores the profound biological and clinical relevance of the 8-gene signature.

To evaluate the predictive performance of the nomogram, we performed time-dependent ROC analysis (Figure 9D). The integrated model yielded excellent AUC values of 0.79, 0.82, and 0.83 for predicting 1-, 2-, and 3-year OS, respectively, markedly outperforming the risk signature alone. This confirms that incorporating clinical variables significantly augments the model’s discriminatory power.

Furthermore, calibration curves were generated to assess the fit quality of the nomogram (Figure 9E). We observed a high degree of concordance between the predicted survival probabilities and the actual observations, demonstrating the model’s excellent calibration. This robust alignment suggests that the model could reliably provide personalized and precise survival predictions for AML patients in real-world clinical settings.

In summary, the integrated nomogram demonstrated exceptional predictive power and clinical utility, representing a highly promising decision-support tool for risk stratification and treatment decision-making in AML patients.

## 4. Discussion

In this study, we developed a multidimensional prognostic model for AML by integrating PCD-related transcriptomic features with clinical variables. Utilizing a deep learning framework, we identified candidate biomarkers reflecting dynamic tumor microenvironmental crosstalk. These features were subsequently refined into a risk signature whose robust prognostic performance was consistently validated across independent external cohorts (GSE10358 and GSE71014), laying the groundwork for a clinically applicable nomogram.

Regarding our methodological design, pre-filtering the initial 1624 PCD genes down to 124 DEGs was essential to remove disease-irrelevant background noise and prevent severe model overfitting, given our limited cohort size (n = 221). Subsequently, the VAE/MLP framework was specifically implemented to address the highly complex and non-linear interactions inherent in AML transcriptomic data. By mapping discrete gene expression profiles into a continuous and probabilistic latent space, the VAE effectively mitigates biological noise. Furthermore, integrating SHAP analysis unlocked the black box of deep learning, achieving a powerful synthesis of superior non-linear modeling capacity and explicit biological transparency.

Through our integrated framework, we ultimately refined these features into an 8-gene prognostic signature (*SORL1*, *PIK3R5*, *RIPK3*, *ELANE*, *GPX1*, *VNN1*, *CD74,* and *IL3RA*). The biological plausibility of this signature is strongly supported by existing literature, encompassing key leukemogenic and microenvironmental processes. Specifically, *RIPK3* (a necroptosis kinase) and *PIK3R5* (a PI3K-AKT regulator) directly modulate AML cell viability and death [65,66]. Microenvironmental interactions and disease progression are reflected by *GPX1* (immunosuppression) [67], *ELANE* (myeloid mutations) [68], and *VNN1* (relapse marker) [69]. Notably, several of these genes represent clinically actionable targets, including *IL3RA* (an AML stem cell marker), *CD74*, and *SORL1* [70,71,72], underscoring the translational potential of our model.

In summary, this study established a robust, multidimensional prognostic model for AML by integrating PCD-related transcriptomic features with clinical variables. The prognostic predictive power of our 8-gene signature was consistently validated across independent external cohorts, yielding highly stable 1-, 2-, and 3-year AUCs > 0.7. Furthermore, a comprehensive nomogram incorporating the risk score and clinical factors (e.g., age and cytogenetic risk) achieved an excellent AUC of 0.83. Ultimately, this extensively validated model provides a clinically actionable framework for prognostic assessment and holds significant promise for guiding personalized therapeutic strategies in AML.

## Data Availability

The LAML dataset was downloaded from the TCGA database (https://portal.gdc.cancer.gov/repository, accessed on 27 February 2025). Additionally, two LAML datasets (GSE10358, n = 92; GSE71014, n = 105) were collected from the GEO database (accessed 21 April 2025).
